# Computer-based tree drawing test in adolescents and adults with depression

**DOI:** 10.3389/fpsyt.2026.1785760

**Published:** 2026-05-08

**Authors:** Hui Jin, Yige Liu, Yunlong Li, Liying Liu, Li Gao, Wei Liu, Yanfei Zhang, Guorui Liu

**Affiliations:** 1 Psychology Research Institute, Nanchang Polytechnic University, Nanchang, China; 2 Institute of Applied Psychology, Jiangsu University, Zhenjiang, China; 3 Department of Psychiatry, Guo Yang County People’s Hospital, Bozhou, China; 4 School of Digital Media & Design Arts, Beijing University of Posts and Telecommunications, Beijing, China; 5 Department of Medical Psychology, Second Affiliated Hospital of Naval Medical University, Shanghai, China; 6 Department of Medical Psychology, No. 905 Hospital of PLA Navy, Shanghai, China

**Keywords:** depression in adolescents, depression in adults, depression screening, depressive disorder, projection test

## Abstract

**Objective:**

To evaluate the value of the computer-based Tree Drawing Test in the auxiliary diagnosis of depressive disorders and to analyze the differences in the performance of adolescent and adult depression patients in the Tree Drawing Projection Test.

**Methods:**

This study was conducted at Guo Yang County People’s Hospital in Anhui, China, and involved a total of 184 participants: 43 adults with depression, 82 adolescents with depression, and 59 healthy controls. The Tree Drawing Test and scale assessments were administered to patients with depressive disorders (adult group and adolescent group) and a control group. Computer image recognition and calculation techniques were used to analyze the results statistically.

**Results:**

Significant differences were observed between the adult depression group and the control group in terms of crown area, trunk area, total area, and HDRS scores (*p* < 0.001). Statistically significant differences were also found between the adult depression group and the adolescent depression group in terms of trunk area (*p* < 0.01), total area (*p* < 0.001), HDRS scores (*p* < 0.001), and HAMA scores (*p* < 0.01). The crown area (*r* = -0.261, *p* < 0.001), trunk area (*r* = -0.154, *p* = 0.037), total area (*r* = -0.285, *p* < 0.001), and HDRS scores in the Tree Drawing Test were significantly correlated.

**Conclusion:**

The computer-based Tree Drawing Test has certain value in the auxiliary diagnosis of depression. Future research should include larger sample sizes and participants from different regions and cultural backgrounds to further validate the generalizability and cultural adaptability of the Tree Drawing Test for depression assessment.

As a type of subjective projection test, the drawing projection test primarily analyzes personality and unconscious content through the analysis of drawing features. Compared to traditional interviews and questionnaires, the drawing projection test has the advantage of reflecting an individual’s inner world through the creative process, without the need for verbal expression, providing a more authentic reflection of psychological states (1). The application of the drawing projection test has a long history and has been widely used in various psychological assessments and auxiliary diagnoses (2). Early studies indicated that the drawing projection test holds significant value in evaluating an individual’s unconscious content, emotional states, and psychological stress.

In clinical practice, the Tree Drawing Test has been widely researched. The subject is instructed to draw a tree, and their psychological state is inferred from the analysis of the tree’s shape, proportions, and other characteristics (3). Existing research has demonstrated that the Tree Drawing Test plays a role in the auxiliary diagnosis of various conditions, including schizophrenia, manic episodes, anxiety disorders, personality disorders, and Alzheimer’s disease (4–8). Regarding the correlation between the Tree Drawing Test and depression, several studies have indicated that certain features in the test are associated with depressive symptoms (9, 10). For example, patients with depression tend to present a monotonous tree shape, with narrower trunks and branches, and may lack vitality, reflecting emotional repression and feelings of helplessness (3, 11, 12). These findings provide preliminary evidence for the potential of the Tree Drawing Test in the auxiliary diagnosis of depression. However, although these studies provide theoretical support for the application of the Tree Drawing Test in depression screening, most have primarily focused on qualitative indicators, which limits the ability to quantify these features. To address this limitation, some researchers have conducted manual measurements of tree drawing indicators (e.g., crown height and width) (13). Building on this approach, subsequent studies introduced computer-assisted methods to quantify these indicators and identified significant differences between individuals with depression and healthy controls across eight metrics, including crown area, trunk area, total tree area, crown height, trunk height, total tree height, crown width, and trunk width (9).

Depression, as a common mental disorder, affects individuals across all age groups globally. Although both adolescents and adults can suffer from depression, there are significant differences between these two groups in terms of the presentation, course, and psychological mechanisms of depression (14). Adolescents are at a critical stage of psychological and physiological development, with emotional regulation, cognitive processing, and self-awareness still maturing. As a result, depressive symptoms in adolescents often manifest as severe mood fluctuations, excessive sensitivity to external pressures, and disconnection from others (15). In contrast, adults, especially those who have undergone long-term social adaptation and stress management, tend to internalize and conceal their emotional expressions. Depressive symptoms in adults are often characterized by low motivation, fatigue, and cognitive difficulties.

These differences are not only reflected in symptomatology but may also extend to emotional regulation mechanisms, social support needs, and coping strategies. During emotional and social development, adolescents may rely more on external support to cope with internal confusion and distress, while adults may develop more complex defense mechanisms and self-regulation capabilities (16). Furthermore, early intervention in adolescent depression is critical, as depression can cause a range of personal, familial, and societal harms (17). The psychological damage may persist into adulthood and lead to a series of issues in family and marital relationships (18, 19). Its development can have profound effects on long-term mental health, social functioning, and academic performance (20, 21). Therefore, understanding the differences between adolescent and adult depression not only aids in accurate diagnosis but also provides a theoretical foundation for developing targeted interventions and treatment plans.

The research team developed the “Tree Drawing Projection Test Analysis Software”. This software automatically calculates data from tree drawings and effectively differentiates between schizophrenia, depression, and control groups (4, 5). However, as the software still requires manual tracing of the tree parts, it remains time-consuming and is not yet suitable for large-scale screening. In this study, the research team upgraded the software by incorporating automated tracing and calculation functions. This study aims to examine the value of computer-automated drawing projection software in the auxiliary diagnosis of depression and to compare the differences in performance on the Tree Drawing Projection Test between adolescent and adult depression patients, with the goal of providing new perspectives and tools for clinical treatment and intervention.

## Methods

This study was conducted at Guo Yang County People’s Hospital in Anhui, China. Participants included individuals recruited from the hospital’s inpatient wards for the depression disorder case groups (depressed adult and depressed adolescent) and healthy controls recruited from the community. The study was approved by the Medical Ethics Committee of Guo Yang County People’s Hospital (approval number: 2023-002). All participants were fully informed about the study’s purpose and procedures before enrollment and provided written informed consent to participate. Recruitment took place from February 2023 to October 2024.

### Inclusion and exclusion criteria

The research design employed a comparative control design, with analyses comparing the adolescent depression group to the adult depression group, and the adult depression group to the adult healthy control group. Purposive sampling was used to select participants for the depression disorder groups, ensuring that all individuals met the DSM-5 diagnostic criteria for depression (22). The adolescent depression group included individuals aged 12–17 years, and the adult depression group included individuals aged 18–65 years. All participants in the depression groups had a Hamilton Depression Rating Scale (HDRS-21) score ≥ 21 (23). Exclusion criteria for the depression groups included a history of schizophrenia, bipolar disorder, or other major psychiatric disorders, as well as cognitive impairment or neurological disorders.

The healthy control group was recruited from the same region and during the same time period as the depression groups. Inclusion criteria for the control group were as follows: no diagnosis of depression based on DSM-5 criteria, no significant psychological symptoms (no positive factors on the Symptom Checklist-90), and no history of psychiatric disorders. The control group was matched to the adult depression group in terms of age and gender.

A total of 184 participants were included in the study, comprising 43 adults with depression, 82 adolescents with depression, and 59 healthy controls. Depressive symptoms were assessed using the Hamilton Depression Rating Scale (HDRS-21). The mean HDRS-21 scores were as follows: Adult depression group (39.63 ± 3.31) and Adolescent depression group (26.73 ± 9.03). Both adolescent and adult participants with depression were undergoing psychiatric treatment at the time of assessment, including the use of antidepressant medications.Specifically, participants underwent the Tree Drawing Test within the first three days of their hospital admission, during which they received only pharmacological treatment (antidepressants) and had not yet started psychological therapy.

### Measures

#### Tree drawing test

Each participant was provided with an A4 sheet of paper and a black or blue-black pen. Participants were instructed to draw under the following guidelines: (1) The drawing projection test is not a test of artistic skills, and no aesthetic quality is required. (2) The drawing projection test is not a still-life drawing; the depiction does not need to resemble objects from nature. (3) If the participant is unable to draw the desired object, they may draw a circle and write a Chinese character inside as a substitute. (4) Before drawing the tree, participants were instructed to close their eyes and meditate for half a minute, visualizing a tree during this time. If no tree appeared during meditation, they were instructed to open their eyes and draw the tree they most wanted to draw. (5) After completing the drawing, participants were asked to write their age and gender on the drawing paper (10), (Note: As cultural differences may affect the evaluation and interpretation of the tree drawing test, this study was limited to participants from mainland China. The cultural background of participants may influence the depiction and symbolic meaning of the tree, and thus the interpretation should consider the potential impact of cultural context on the drawing content (24, 25)).

#### Epson high-resolution scanner (DS-1630)

This study used the Epson High-Resolution Scanner (DS-1630) to scan the tree drawings and store the scanned images on a computer.

#### DSM-5

The Diagnostic and Statistical Manual of Mental Disorders (DSM-5), published by the American Psychiatric Association (APA), is the most commonly used guide for diagnosing mental disorders in the United States and internationally. The first edition was published in 1952, and the fifth edition was released on May 23, 2013 (22).

#### Tree drawing projection test software

**Figure 1:**
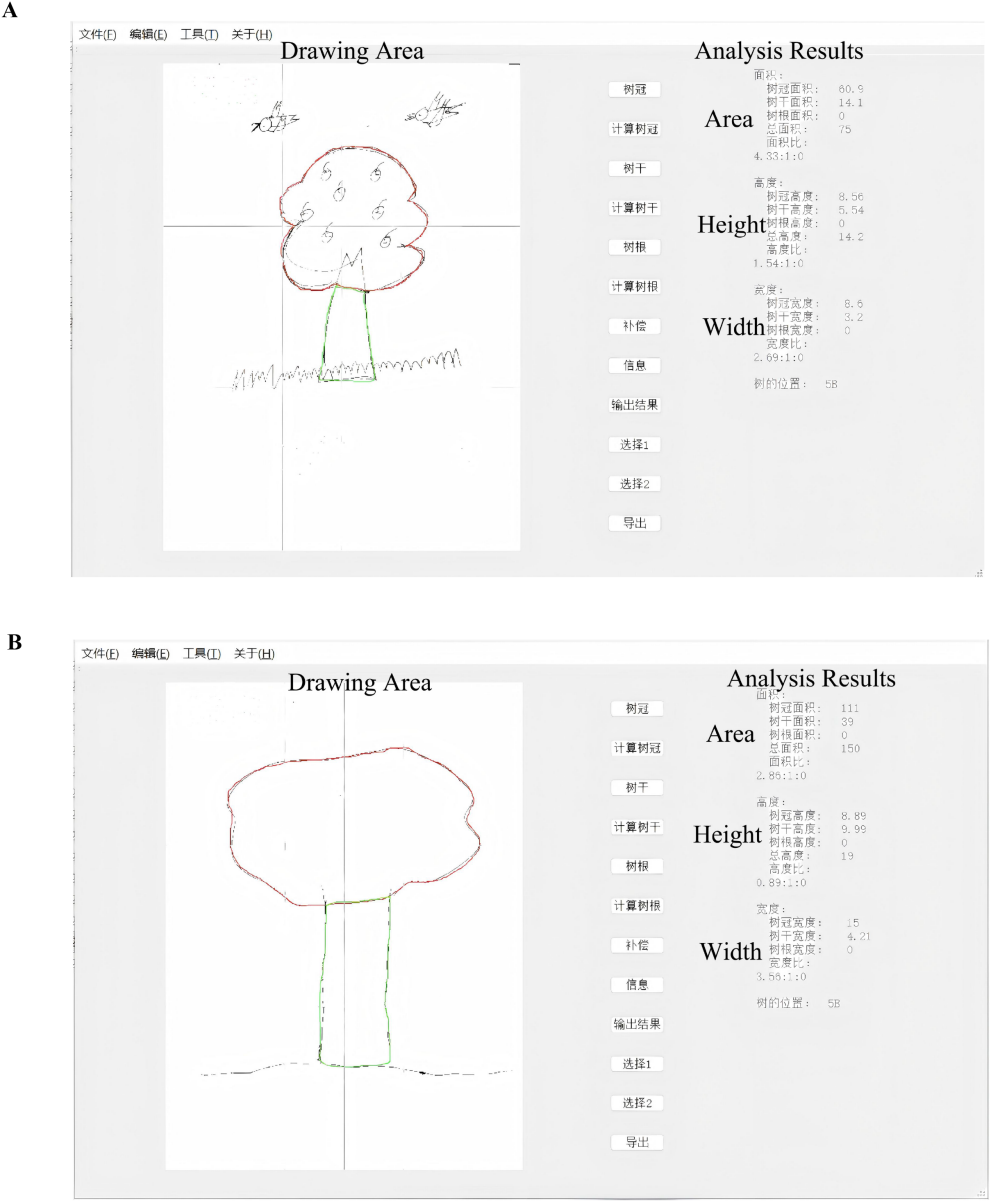
**(A)** Anonymized tree drawings from depressed adults. **(A)** Screenshot of the software’s operational interface during the tree drawing analysis. The interface displays the Drawing Area on the left, which shows the imported tree drawing image, with key structural components (e.g., crown, trunk, and roots) identified through contour detection. The Analysis Results panel on the right presents the quantitative measurements for each component. The Area section on the right outputs the area results for the crown, trunk, and roots. The Height section displays the height results for these components, while the Width section shows their width results. The software automatically calculates and displays these measurements based on pixel values, which are converted into standardized units (cm or cm²) through calibration parameters. Note: The software uses automated image processing to reduce subjective bias, offering semi-automated analysis that provides objective quantification of the tree drawing’s key features. **(B)** Anonymized tree drawings health controls. **(B)** Screenshot of the software’s operational interface during the tree drawing analysis. The interface displays the Drawing Area on the left, which shows the imported tree drawing image, with key structural components (e.g., crown, trunk, and roots) identified through contour detection. The Analysis Results panel on the right presents the quantitative measurements for each component. The Area section on the right outputs the area results for the crown, trunk, and roots. The Height section displays the height results for these components, while the Width section shows their width results. The software automatically calculates and displays these measurements based on pixel values, which are converted into standardized units (cm or cm²) through calibration parameters. The software uses automated image processing to reduce subjective bias, offering semi-automated analysis that provides objective quantification of the tree drawing’s key features.

The Software used in this study was developed based on our previous work and further upgraded with automated tracing and calculation functions (10). The software processes scanned images of tree drawings and performs semi-automated image analysis. The language of the software is Chinese. First, images are imported into the system following standardized scanning procedures (Epson DS-1630, consistent resolution). The software then identifies key structural components of the tree drawing (e.g., crown, trunk, and roots) through contour detection and region segmentation (see **Figures 1A, B**).

Following segmentation, quantitative features are extracted, including length, width, height, and area of each component. All measurements are calculated based on pixel values and converted into standardized units (cm or cm²) using calibration parameters. The automated calculation reduces subjective bias associated with manual measurement.

The reliability and validity of the software have been evaluated in previous studies, where automated measurements showed high agreement with manual measurements and demonstrated good discriminative ability between clinical and control groups. In the present study, all images were processed using the same standardized workflow to ensure consistency.

To minimize bias, image processing and statistical analyses were conducted using anonymized data. The analysts were not involved in participant recruitment or clinical diagnosis.

### Statistical methods

Data were organized and analyzed using SPSS 26.0 statistical software. Continuous variables were expressed as mean ± standard deviation (x ± s), and categorical variables were expressed as frequency (n). The main statistical tests used included independent samples t-test, chi-square analysis, and Spearman correlation analysis. The normality of the data was assessed using the Shapiro-Wilk test. For variables that did not follow a normal distribution, data transformation or non-parametric tests were employed. All statistical tests were conducted with a significance level set at 0.05. Effect sizes (Cohen’s d) were calculated for group comparisons to assess the magnitude of differences. Independent samples t-tests were used for normally distributed variables, while non-parametric tests were applied when normality assumptions were violated.

### Sample size and statistical power analysis

Assuming an effect size of 0.5 (medium effect), a significance level of 0.05, and a statistical power of 80%, the sample size calculation for the t-test indicated that the minimum required sample size would be 64 participants per group (total 128 participants). A total of 125 participants were included in the case group and 59 in the control group, meeting the statistical power requirements for the study design.

## Results

A total of 82 adolescents with depression were enrolled in the study, including 38 males and 44 females, with an average age of 14.70 ± 1.89 years. The adult depression group consisted of 43 participants, including 20 males and 23 females. The healthy control group included 59 participants, with 29 males and 30 females (see **Table 1**).

**Table 1:** Sociodemographic and clinical profile of participants.

Variable	Depressed adolescents	Depressed adults	Healthy controls
Gender			
Male	38 (46.3%)	20 (46.5%)	29 (49.2%)
Female	44 (53.7%)	23 (53.5%)	30 (50.8%)
Age	14.70 ± 1.89	45.02 ± 15.21	43.95 ± 11.47
Education level			
Primary School	0 (0.0%)	2 (4.7%)	3 (5.1%)
Junior High School	19 (23.2%)	6 (14.0%)	5 (8.5%)
High School	63 (76.8%)	30 (69.8%)	45 (76.3%)
College or Higher	0 (0.0%)	5 (11.6%)	6 (10.2%)
HDRS scores			
19-22	20 (24.4%)	3 (7.0%)	–
≥ 23	62 (75.6%)	40 (93.0%)	–

Participants in the depression groups all had a Hamilton Depression Rating Scale (HDRS-21) score ≥ 21.

### Comparison of tree drawing indices and HDRS scores between the adult depression group and healthy controls

**Table 2** indicates that the adult depression group had smaller crown area (45.18 ± 4.15, *p* < 0.001), trunk area (8.87 ± 1.05, *p* < 0.001), and total area of the tree drawing (56.71 ± 4.20, *p* < 0.001) compared to the healthy control group, and a higher HDRS score (39.63 ± 3.31, *p* < 0.001) than the healthy control group, with statistically significant differences.

**Table 2:** Comparison of tree drawing indices and HDRS scores between the depressed adults and healthy controls.

Variable	Depressed adults	Healthy controls	*t*	*p*
Crown Area (cm²)	45.18 ± 4.15	90.84 ± 5.12	-4.172	< 0.001^***^
Trunk Area (cm²)	8.87 ± 1.05	19.38 ± 1.34	-4.005	< 0.001^***^
Total Area (cm²)	56.71 ± 4.20	114.53 ± 9.53	-4.445	< 0.001^***^
HDRS	39.63 ± 3.31	4.80 ± 2.83	57.097	< 0.001^***^

***p < 0.001.

### Comparison of tree drawing indices and HDRS/HAMA scores between the adolescent and adult depression groups

**Figure 2:**
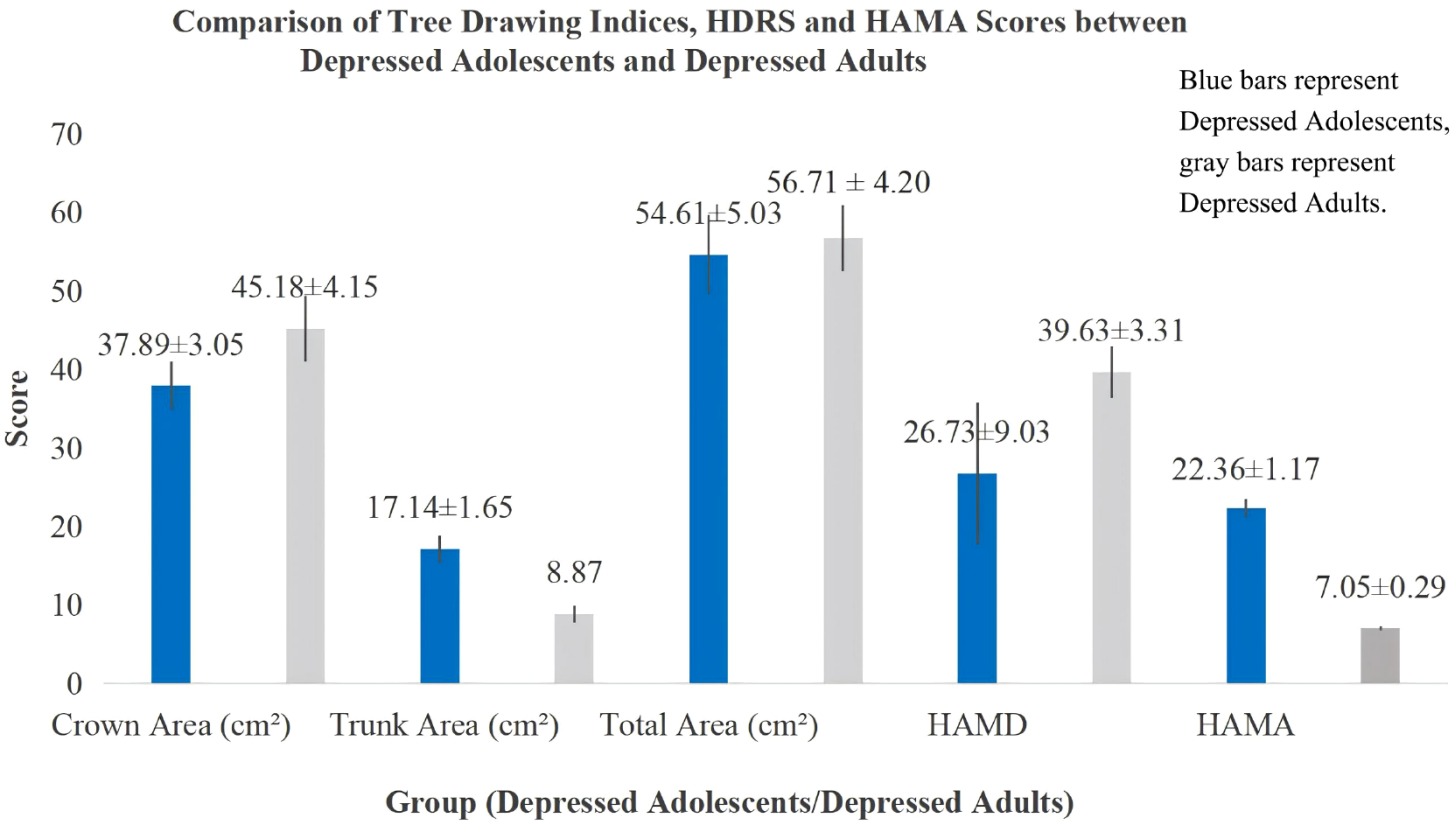
Comparison of tree drawing indices, HDRS, and HAMA scores between depressed adolescents and depressed adults.

**Figure 3:**
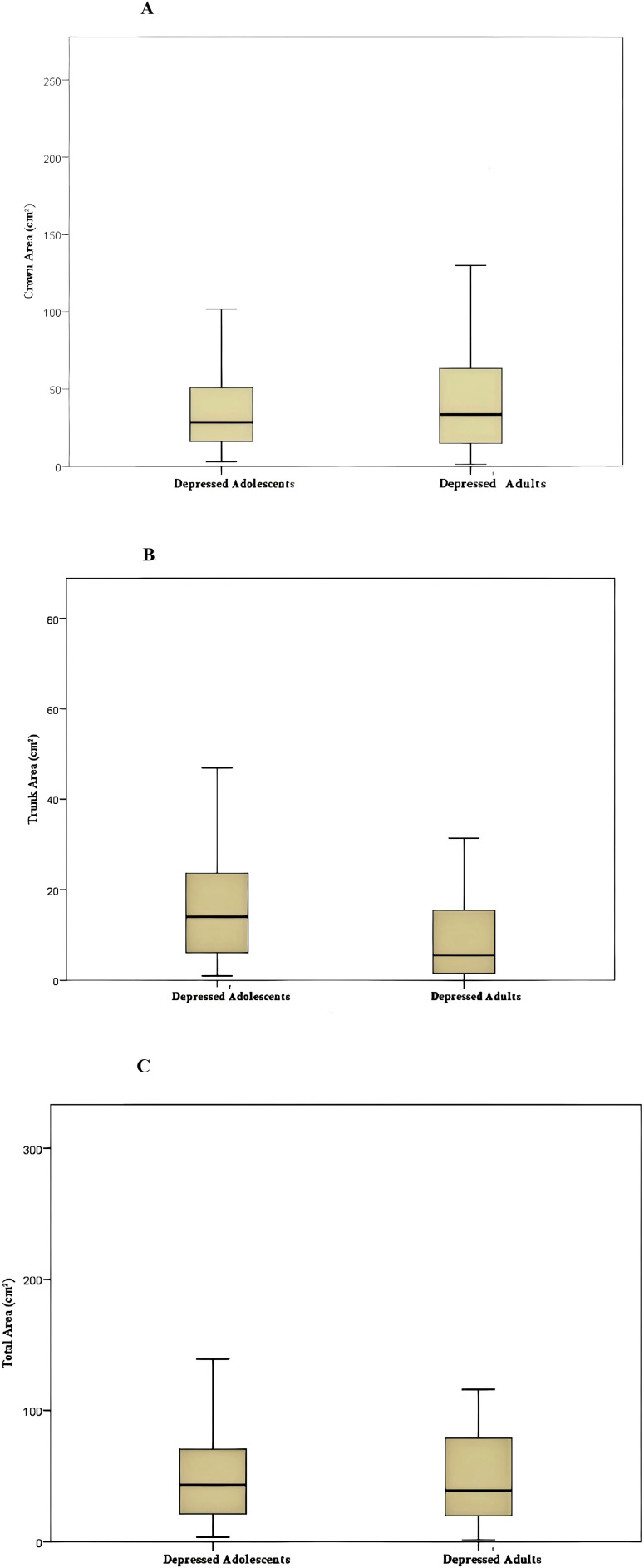
**(A)** Boxplot of crown area across diagnostic groups. **(B)** Boxplot of trunk area across diagnostic groups. **(C)** Boxplot of total area across diagnostic groups. Boxplot of crown area, trunk area, and total area across depressed adolescent and Depressed adult. The box represents the interquartile range, the horizontal line indicates the median, and whiskers represent the range of non-outlier values.

The crown area in the adolescent depression group (37.18 ± 3.05) was smaller than that in the adult depression group (45.18 ± 4.15), though the difference was not statistically significant (*p* = 0.301) (see, **Table 3**; **Figures 2**, **3**). The trunk area and HAMA score were higher in the adolescent depression group compared to the adult depression group (*p* < 0.01), while the total area and HDRS score were smaller in the adolescent depression group compared to the adult depression group (*p* < 0.001) (see, **Table 3**; **Figure 2**).

**Table 3:** Comparison of tree drawing indices, HDRS, and HAMA scores between the depressed adolescents and depressed adults.

Variable	Depressed adolescents	Depressed adults	*t*	*p*
Crown Area (cm²)	37.89 ± 3.05	45.18 ± 4.15	-1.038	0.301
Trunk Area (cm²)	17.14 ± 1.65	8.87 ± 1.05	3.410	0.001^**^
Total Area (cm²)	54.61 ± 5.03	56.71 ± 4.20	-4.445	< 0.001^***^
HDRS	26.73 ± 9.03	39.63 ± 3.31	-9.040	< 0.001^***^
HAMA	22.36 ± 1.17	7.05 ± 0.29	9.409	< 0.001^***^

**p < 0.01, ***p < 0.001.

### Correlation between tree drawing indices and HDRS scores

As shown in **Table 4**, the crown area (*r* = -0.261, *p* < 0.001), trunk area (*r* = -0.154, *p* = 0.037), total area (*r* = -0.285, *p* < 0.001) of the tree drawing, and HDRS scores were significantly correlated.

**Table 4:** Correlation between tree drawing indices and HDRS scores.

Variable	HDRS	
	*r*	*p*
Crown Area (cm²)	-0.261	< 0.001***
Trunk Area (cm²)	-0.154	0.037
Total Area (cm²)	-0.285	< 0.001***

***p < 0.001.

Effect size analysis for the comparisons of Tree Drawing Indices and HDRS scores between the adult and control groups, as well as the comparisons of Tree Drawing Indices, HDRS, and HAMA scores between the adolescent and adult groups, showed that most group differences were associated with large to very large effects (Cohen’s d > 0.8), indicating strong practical significance of the findings.

## Discussion

The results of this study are consistent with previous findings, which showed that individuals with adult depression had smaller crown area, trunk area, and total area compared to healthy controls (9). In this study, the crown area, trunk area, total area, and HDRS scores were significantly correlated (*p* < 0.001). These results suggest that the Tree Drawing Test holds potential value in the auxiliary diagnosis of depression. By employing computer-assisted shape tracing and data computation, this method significantly shortens the testing time and enhances the accuracy of the results, making it valuable for depression screening. In addition, computer-based software analysis offers relatively standardized procedures and reduces reliance on professional experience in drawing interpretation, thereby providing a technical basis for the broader implementation of this test across different institutions.

This study aimed to compare the performance differences in the Tree Drawing Test between adolescent and adult depression patients and to explore potential age-related differences in emotional expression and psychological mechanisms. The results revealed that while no significant differences were found in the crown area of the tree drawings between adolescents and adults, significant differences were observed in trunk area, total area, HDRS, and HAMA scores.

Regarding the trunk area, the adolescent group had a larger trunk area than the adult group. In the Tree Drawing Test, the trunk is considered to reflect an individual’s emotions and is seen as a symbol of inner self (6). Previous studies have indicated that individuals with depression tend to draw smaller trunks compared to healthy controls, which is thought to reflect characteristics such as low mood and negative pessimism. The larger trunk area in the adolescent group suggests that the emotional expression of depression in adolescents is more likely to be unstable, and the degree of emotional depression may be less severe than in adults, as reflected in the lower HDRS scores for the adolescent group. The higher HAMA score in adolescents suggests that adolescent depression is more likely to be accompanied by anxiety symptoms. Research has suggested that the core diagnostic symptom of depression in adolescents — subtle mood depression — is often missed, possibly because adolescent depression is more likely to manifest as irritability and emotional instability (14).

In terms of quantifying depressive and anxiety symptoms, significant differences were found between the adolescent and adult groups. The adolescent group had lower depression scores, while the adult group exhibited more pronounced anxiety symptoms. These results may reflect the emotional immaturity of adolescents in emotional cognition and coping, leading them to externalize their emotional distress or exhibit behavioral problems when facing depressive symptoms (26). In contrast, adult depression patients may be more likely to internalize their negative emotions, which manifest as low motivation and cognitive impairment. This highlights the need for personalized treatment strategies for depression patients of different age groups, especially in terms of emotional regulation and cognitive processing. Moreover, the Tree Drawing Test combined with computer-assisted analysis features ease of operation and a high level of quantitative output. In addition to clinical outpatient settings, it may also have potential applications in school-based mental health screening, community mental health services, and preliminary psychological assessment in primary healthcare institutions. The use of standardized software-based analysis procedures may help reduce subjective variability in manual evaluations and improve screening efficiency.

One of the drawbacks of drawing projection tests is that there are cultural differences in interpreting the results. Although some studies have shown that drawing projection tests have high reliability and validity, such as the research by Wan Chao et al. (2014), which found that the test-retest correlation of the tree drawing test at different time intervals ranged from 0.570 to 0.733 and 0.341 to 0.713 (P < 0.05); the Kendall concordance coefficient among three raters was 0.491 to 0.626 (P < 0.05); and the correlation between relevant indicators in the drawing test and corresponding indicators in the 16PF, SDS, and SAS tests was significant (P < 0.05) (27). However, other studies have shown that the interpretation of the same drawing indicator can differ across cultures, raising questions about the accuracy of the drawing projection test (6). The main reason for this may be due to differences in sample characteristics, cultural backgrounds, and other factors (24, 28, 29). Participants’ cultural backgrounds may affect the form and symbolic meaning of the objects they draw. Therefore, when interpreting the test results, the potential influence of cultural context on the content of the drawings should be taken into account (30). The present study contributes to this field by applying a computer-based quantitative approach, which may reduce subjectivity; however, further validation is needed to establish its robustness across diverse populations. Additionally, both adolescent and adult participants with depression were receiving psychiatric treatment at the time of testing, including medications such as antidepressants. This concurrent treatment represents a potential confounding variable, as the effects of medication could influence the results of the Tree Drawing Test. Although the inclusion of participants under treatment reflects real-world clinical settings, it is important to note that the treatment status could have impacted the results.

Although the findings of this study have certain clinical reference value, there are several limitations that should be considered. First, the sample size was relatively small, which may limit the generalizability of the results. Second, due to the convenience of sample collection, a healthy adolescent control group was not included in the present study. Consequently, direct comparisons of tree drawing characteristics between adolescents with depression and healthy adolescents were not possible, which may have limited the interpretation of tree drawing features in the adolescent population. Future studies should include a healthy adolescent control group to further explore the potential value of the Tree Drawing Test as an auxiliary tool for the diagnosis and severity assessment of depressive disorders in adolescents. Third, all participants were recruited from a single hospital, with a relatively homogenous geographic and cultural background, which may not adequately represent a broader population of adolescents and adults, particularly those from different regional or ethnic backgrounds (28, 29). Furthermore, the software system used in this study has primarily been validated in hospital settings. Its applicability across different institutions, equipment conditions, and populations still requires further investigation. Fourth, potential selection bias cannot be excluded, as participants were recruited from clinical and community settings, which may not fully represent the general population. In addition, the sample sizes between groups were not fully balanced, which may affect statistical power and the stability of the findings. Future studies should aim to include larger and more diverse samples, incorporate multicenter designs, and adopt longitudinal approaches to better understand causal relationships and developmental changes over time. In addition, future research should further evaluate the applicability of this method in different real-world settings, such as schools, community mental health services, and primary healthcare institutions.

In conclusion, the Tree Drawing Test based on computer analysis technology shows promising effectiveness in the auxiliary diagnosis of depression. Future research should include larger sample sizes and participants from diverse regions and cultural backgrounds to further validate the generalizability and cultural adaptability of the Tree Drawing Test for depression assessment. In addition, future studies may further explore the potential applications of this software in remote psychological assessment, digital mental health screening, and mental health management platforms, thereby enhancing its accessibility and practical value in broader real-world settings. But, it is important to clarify that the Tree Drawing Test in this study is primarily an associative tool, reflecting certain psychological characteristics related to depression, rather than providing definitive diagnostic capabilities. The results suggest that the test may help identify signs of depression, but it cannot be used as a standalone diagnostic tool. The Tree Drawing Test may have practical applications in screening for depression, particularly in settings where access to more formal diagnostic tools is limited, such as in schools, primary care clinics, or community mental health centers. While it cannot replace comprehensive clinical assessments, it may serve as a supportive tool to help identify individuals who may require further evaluation or intervention. When compared with established diagnostic tools, such as structured clinical interviews or validated depression scales (e.g., Hamilton Depression Rating Scale [HDRS]), the Tree Drawing Test offers a complementary approach to assessing depressive symptoms. Unlike structured interviews, which provide in-depth diagnostic evaluation, the Tree Drawing Test is a more rapid and less resource-intensive method. Future research should explore the feasibility of implementing this method in routine screenings, especially in resource-limited settings.

## Data Availability

The original contributions presented in the study are included in the article/supplementary material. Further inquiries can be directed to the corresponding authors.
